# Biomarker Analysis of Orally Dosed, Dual Active, Matrix Metalloproteinase (MMP)-2 and MMP-9 Inhibitor, AQU-118, in the Spinal Nerve Ligation (SNL) Rat Model of Neuropathic Pain

**DOI:** 10.3390/ijms20040811

**Published:** 2019-02-14

**Authors:** Mei Yee Kwan, Anthony Choo, Taleen Hanania, Afshin Ghavami, Jose Beltran, John Shea, Amidi Barboza, Andrew Hu, Marcie Fowler, Venugopal Rao Neelagiri, Irving Sucholeiki

**Affiliations:** 1 PsychoGenics Inc., 215 College Road, Paramus, NJ 07652, USA; Mei.Kwan@psychogenics.com (M.Y.K.); amchoo@gmail.com (A.C.); Taleen.Hanania@psychogenics.com (T.H.); Afshin.Ghavami@psychogenics.com (A.G.); Jose.Beltran@psychogenics.com (J.B.); John.Shea@psychogenics.com (J.S.); Amidi.Barboza@psychogenics.com (A.B.); andreweiy2000@yahoo.com (A.H.); 2 United States Army of Surgical Research, Joint Base San Antonio (JBSA), Fort Sam Houston, TX 78234, USA; Marcie.fowler.phd@gmail.com; 3 Aquilus Pharmaceuticals Inc., 3H Gill Street, Suite 300, Woburn, MA 01801, USA; neelagiriv@yahoo.com

**Keywords:** matrix metalloproteinase, MMP-2, MMP-9, inhibitor, allodynia, caspase-3, neuropathic, pain, dorsal root ganglion, spinal nerve ligation

## Abstract

There is an unmet medical need for the development of non-addicting pain therapeutics with enhanced efficacy and tolerability. The current study examined the effects of AQU-118, an orally active inhibitor of metalloproteinase-2 (MMP-2) and MMP-9, in the spinal nerve ligation (SNL) rat model of neuropathic pain. Mechanical allodynia and the levels of various biomarkers were examined within the dorsal root ganglion (DRG) before and after oral dosing with AQU-118. The rats that received the SNL surgery exhibited significant mechanical allodynia as compared to sham controls. Animals received either vehicle, positive control (gabapentin), or AQU-118. After SNL surgery, the dorsal root ganglion (DRG) of those rats dosed with vehicle had elevated messenger RNA (mRNA) expression levels for MMP-2, IL1-β & IL-6 and elevated protein levels for caspase-3 while exhibiting decreased protein levels for myelin basic protein (MBP) & active IL-β as compared to sham controls. Rats orally dosed with AQU-118 exhibited significantly reduced mechanical allodynia and decreased levels of caspase-3 in the DRG as compared to vehicle controls. Results demonstrate that oral dosing with the dual active, MMP-2/-9 inhibitor, AQU-118, attenuated mechanical allodynia while at the same time significantly reduced the levels of caspase-3 in the DRG.

## 1. Introduction

The population suffering from neuropathic pain is large; neuropathic pain is a significant problem for patients and the healthcare system. This type of pain is difficult to treat effectively with currently available pharmacological agents, in part due to the fact that the aetiologies that drive the neuropathic pain states are varied, complex, and not well-understood. Indeed, many of the currently approved neuropathic pain medications, and in particular anticonvulsants such as gabapentin and pregabalin, do not have a direct effect on the pathophysiology of the disorder but rather modulate neurotransmission [1]. Since only one in four patients with neuropathic pain experiences greater than a 50% reduction in pain following treatment, novel therapeutic approaches are an urgent priority [2]. One tactic for treating neuropathic pain is through the inhibition of specific protein targets involved in the initiation or maintenance of pain sensitization within the spinal cord.

Surgically-induced rodent models of neuropathic pain cause the levels of various inflammatory proteins such as cytokines and, in particular, certain matrix metalloproteinases (MMPs), to become elevated within the spinal nerves [3,4,5,6,7,8,9]. MMPs are zinc-dependent endopeptidases belonging to a larger family of proteases known as the metzincin superfamily. MMPs are involved in degrading the extracellular matrix as well as processing of growth factors, cytokines, chemokines, adhesion molecules, and a variety of other enzymes. Increased expression of MMPs or an imbalance of different MMPs has been observed in a variety of pathological conditions, including various neurodegenerative diseases [10,11].

It has been postulated that in injured dorsal root ganglion (DRG) primary sensory neurons, MMP-9 can induce early neuropathic pain via interleukin-1β cleavage and microglia activation, and MMP-2 induces delayed neuropathic pain via IL-1β cleavage and astrocyte activation [12]. MMP-9 also promotes Schwann cell-mediated myelin basic protein (MBP) degradation and macrophage infiltration in the spinal nerve and activation of astrocytes in the spinal cord [13]. Based upon this evidence, we sought to develop an orally active MMP-2 and MMP-9 inhibitor to treat neuropathic pain. Our first compound in this arena is AQU-118, a potent small molecule inhibitor of both MMP-2 and MMP-9 with an in vitro IC50 of 3 nM and 9 nM, respectively [14]. AQU-118 attenuates neuropathic pain behaviors in the SNL and chronic constriction injury of the infraorbital nerve (CCI-IoN) rodent models as well as in the naloxone precipitated morphine withdrawal rodent model [14,15]. While these models demonstrated the analgesic efficacy of AQU-118 in neuropathic pain, they did not address the mechanistic or biochemical correlates associated with this activity. The present study analyzes the effects of AQU-118 on mechanical allodynia in the SNL-rat model and then identifies underlying molecular effects of oral dosing with AQU-118 on various inflammatory biomarkers associated with neuropathic pain.

Although many biomarkers are associated with neuropathic pain, we focused on those whose expression and/or protein levels have been reported to change within the dorsal root ganglion (DRG) or dorsal horn following spinal nerve injuries [12,13,16,17,18]. We examined transcript expression levels of MMP-2, MMP-9, IL-1β, IL-6 and protein levels of MBP (20 kDa and 15 kDa molecular weight bands), the pro and active forms of IL-1β, IL-6 and caspase-3 from rat DRG samples. We first determined if any protein or mRNA expression changes were related to the spinal nerve ligation (SNL) surgery by comparing DRG samples from rats who received the SNL with control animals that underwent a sham surgery. Any biomarker(s) that were observed to significantly change in the DRG after SNL-surgery were then examined further to determine if they would return to pre-SNL levels (i.e., sham) upon oral dosing with AQU-118 and its corresponding reduction in mechanical allodynia. The ultimate goals were to better understand the mechanism of action for the antiallodynic effects of AQU-118 and to find biomarkers that could be used in a clinical setting to help monitor the effectiveness of AQU-118. This study presents for the first time biomarker data for an orally administered, dual active MMP-2/MMP-9 inhibitor in the SNL rat model for neuropathic pain. These results further validate the oral effectiveness of AQU-118 and support its continued preclinical development and future clinical testing as a novel treatment for general neuropathic pain.

## 2. Results

### 2.1. Attenuation of von Frey Mechanical Allodynia by Oral Administration of AQU-118 in the Spinal Nerve Ligation (SNL)-Rat Model

**Figure 1:**
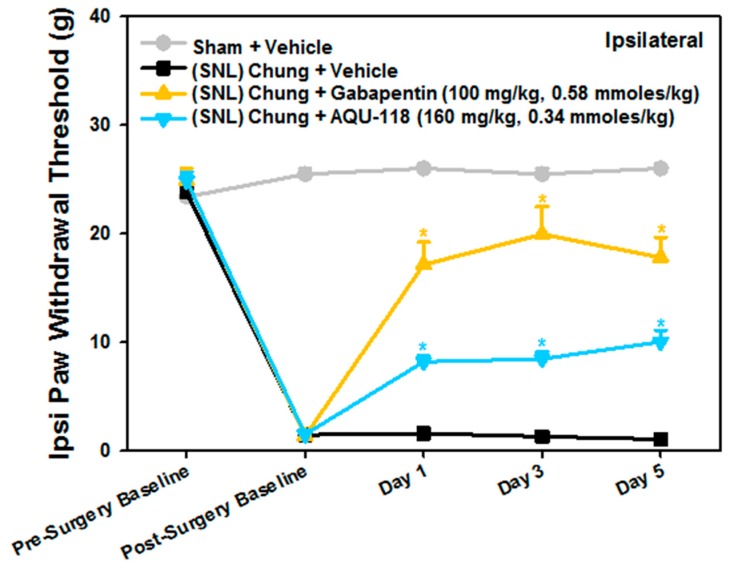
Mechanical Response Threshold: Spinal Nerve Ligation (SNL) Chung Model (Chung), Ipsilateral (Ipsi) Paw Withdrawal. Paw withdrawal thresholds following SNL-surgery for ipsilateral hind paws. Data are presented as mean ± SEM. * *p* < 0.05 vs. (SNL) Chung + Vehicle group on the same day. Post-Surgery Baseline was performed 15 days after SNL-surgery.

**Figure 2:**
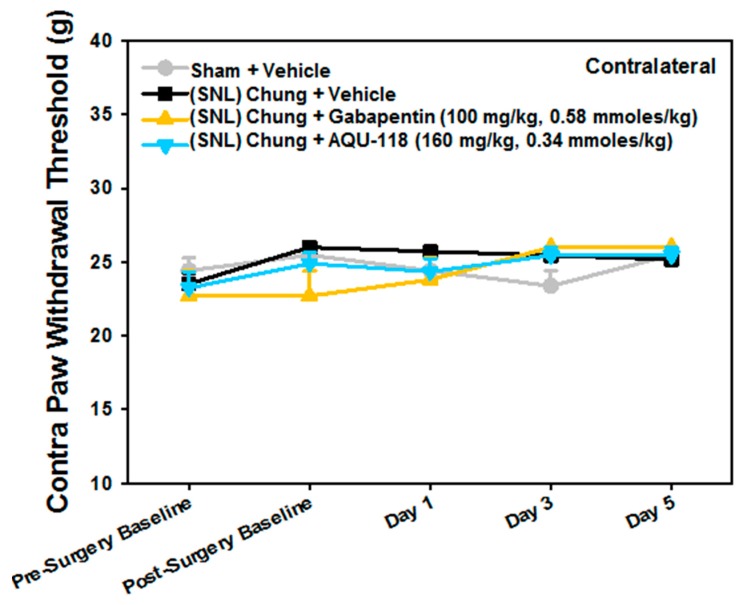
Mechanical Response Threshold: Spinal Nerve Ligation (SNL) Chung Model (Chung), Contralateral (Contra) Paw Withdrawal. Contralateral paw withdrawal thresholds following surgery and administration of AQU-118. Data are presented as mean ± SEM.

Due to the need for sufficient spinal tissue (i.e., DRG) to perform both the gene expression and protein biomarker work, the SNL-rat model studies were designed to incorporate higher numbers of rodents among certain groups than would typically be needed for measuring only mechanical allodynia. Except for the positive control (*n* = 10, gabapentin, 100 mg/kg, 0.58 mmoles/kg), a larger number of rodents were used for the sham (*n* = 20), vehicle (0.5% methyl cellulose, *n* = 40) and drug groups (*n* = 20, AQU-118, 160 mg/kg, 0.34 mmoles/kg) (Table 1). Two weeks after surgery, rats with L-5 spinal nerve ligation displayed significant mechanical allodynia as compared to pre-operative testing (Figure 1). Oral dosing of AQU-118 beginning on day 1 caused an increase in the paw withdrawal threshold (PWT) as compared to the vehicle control group (Figure 1). No statistically significant effect on contralateral PWT was observed with oral dosing of AQU-118 which was comparable to both the vehicle and positive control (gabapentin) arms (Figure 2).

### 2.2. Transcript Expression Changes in the DRG 20 Days after SNL-Surgery (D5) between Vehicle and Sham Groups

**Figure 3:**
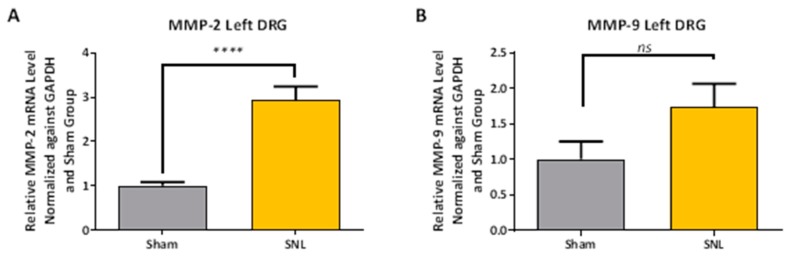
MMP2 (**A**) and MMP9 (**B**) mRNA expression levels in ipsilateral DRG for sham and SNL vehicle groups. MMP-2 mRNA expression levels were found to be significantly elevated after SNL-surgery while MMP-9 mRNA was found to be not significant. Data presented as the average from *n* = 10 per group ± SEM and analyzed with a two-tailed unpaired t-test (ns, not significant; ****, *p* < 0.0001).

**Figure 4:**
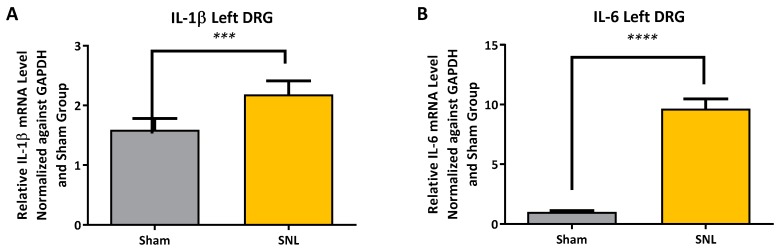
IL-1β (**A**) and IL-6 (**B**) mRNA expression levels in ipsilateral DRG for sham and SNL vehicle groups. Both IL-1β and IL-6 mRNA expression levels were found to be significantly elevated after SNL-surgery in vehicle group compared to sham. Data presented as the average from *n* = 10 per group ± SEM and analyzed a with two-tailed unpaired t-test. (***, *p* < 0.001; ****, *p* < 0.0001).

**Figure 5:**
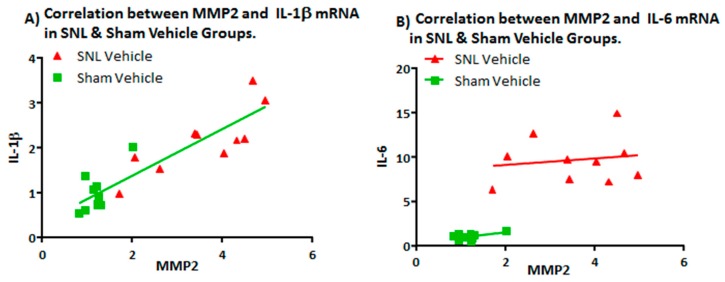
Correlation between MMP2 and (**A**) IL-1β mRNA and (**B**) IL-6 mRNA expression levels in left DRG of SNL & sham Vehicle groups. Pearson correlation coefficients (with 95% confidence) were calculated for sham vehicle (*r* = 0.7372; two-tailed *p* value = 0.015) and SNL vehicle (*r* = 0.8135; two-tailed *p* value = 0.0042) for MMP2 vs IL-1β mRNA. Similar analysis of MMP2 vs. IL-6 mRNA showed no clear correlation with IL-6 transcript level: sham vehicle (*r* = 0.4912; two tailed *p* value = 0.1494) and SNL vehicle (*r* = 0.1622; two tailed *p* value = 0.6543).

Comparisons of MMP-2, MMP-9, IL-1β and IL-6 mRNA expression levels in the left (ipsilateral) DRG between vehicle and sham groups were measured and all were found to be significantly elevated except for MMP-9 (Figure 3 and Figure 4). An apparent correlation was observed between the mRNA expression levels of MMP-2 and IL-1β (Figure 5). However, no such correlation was observed between MMP-2 and IL-6 mRNA levels (Figure 5).

### 2.3. Protein Level Changes in the DRG 20 Days after SNL-Surgery (D5) between Vehicle and Sham Groups

**Figure 6:**
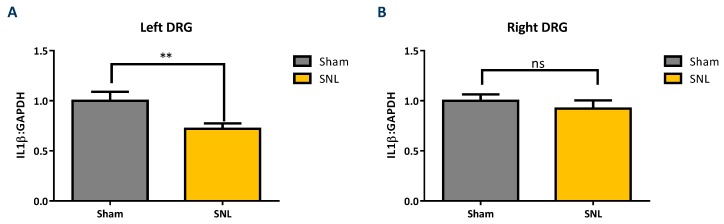
Comparison of cleaved IL-1β protein level in ipsilateral and contralateral DRG for sham and vehicle groups. Cleaved, active IL-1β protein levels in left (ipsilateral) DRG (**A**) significantly decreased in the vehicle group (*n* = 10) compared to the sham (*n* = 10). (**B**) No difference between the sham (*n* = 10) and vehicle group (*n* = 10) in right (contralateral) DRG. Data presented as the average ± SEM. An unpaired t-test was performed: ns, not significant; **, *p* < 0.01.

**Figure 7:**
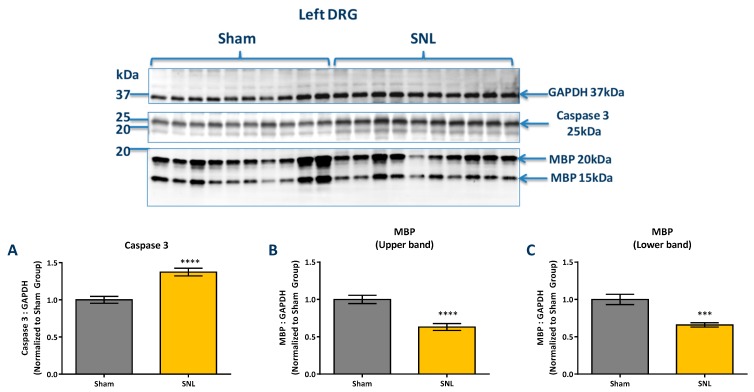
Comparison of Caspase-3 and MBP protein levels in in the ipsilateral DRG for sham and vehicle groups. (**A**) Caspase-3 protein levels (via western blot) in the left (ipsilateral) DRG significantly increased in the vehicle group compared to the sham. (**B**) MBP levels (via western blot) significantly decreased for both the upper band (20 kDa) and (**C**) lower band (15 kDa) in the vehicle group of the left (ipsilateral) DRG as compared to the sham. Protein level is presented normalized to GAPDH and relative to the sham group. Data presented as the average ± SEM. An unpaired t-test was performed: ***, *p* < 0.001; ****, *p* < 0.0001.

IL-1β (Pro and cleaved form), MBP, IL-6 and caspase-3 protein levels were measured via western blotting of tissue obtained from the left (ipsilateral) DRG and compared between sham and vehicle groups. All protein targets were normalized to glyceraldehyde-3-phosphate dehydrogenase (GAPDH) protein levels. The results show that cleaved IL-1β was found to significantly decrease in the ipsilateral DRG of the vehicle group as compared to sham while no significant changes were observed in contralateral DRG (Figure 6). Caspase-3 was found to significantly increase in the ipsilateral DRG while both the higher and lower molecular weight bands of MBP were found to significantly decrease in the vehicle group as compared to the sham (Figure 7). The pro forms of IL-1β and IL-6 were not detected in the DRG by western blotting.

### 2.4. Protein Level Changes in the DRG 20 Days after SNL-Surgery (D5) between AQU-118 and Vehicle Groups

**Figure 8:**
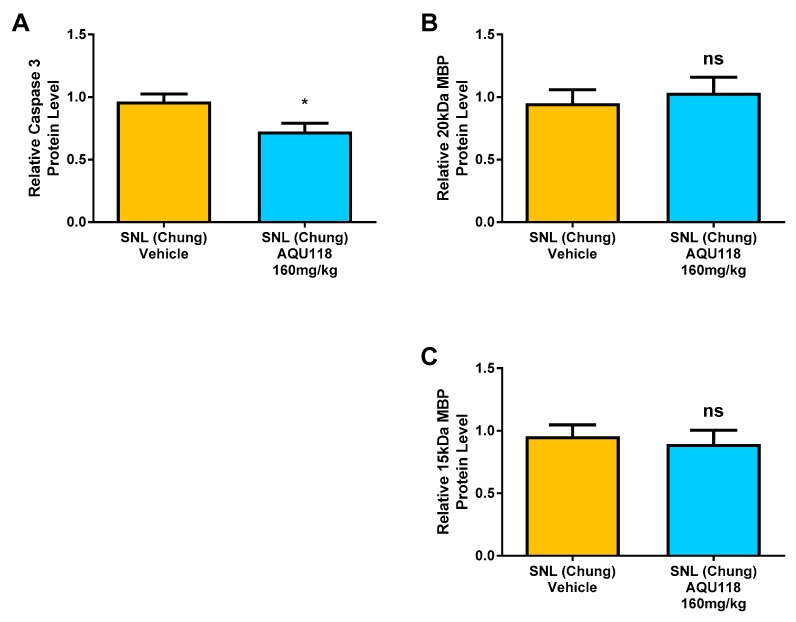
Comparison of caspase-3 and MBP in the ipsilateral DRG for vehicle and AQU-118 groups. (**A**) Caspase-3 protein levels in the left (ipsilateral) DRG significantly decreased in the AQU-118 group (*n* = 20) compared to the vehicle (*n* = 20). (**B**) There were no significant changes in MBP levels in both the upper band (20 kDa) and (**C**) lower band (15 kDa) in AQU-118 group (*n* = 20) of the left (ipsilateral) DRG as compared to the vehicle group (*n* = 20). Protein level is presented normalized to GAPDH and relative to the vehicle group. Data presented as the average ± SEM. An unpaired t-test was performed: ns, not significant; *, *p* < 0.05.

**Figure 9:**
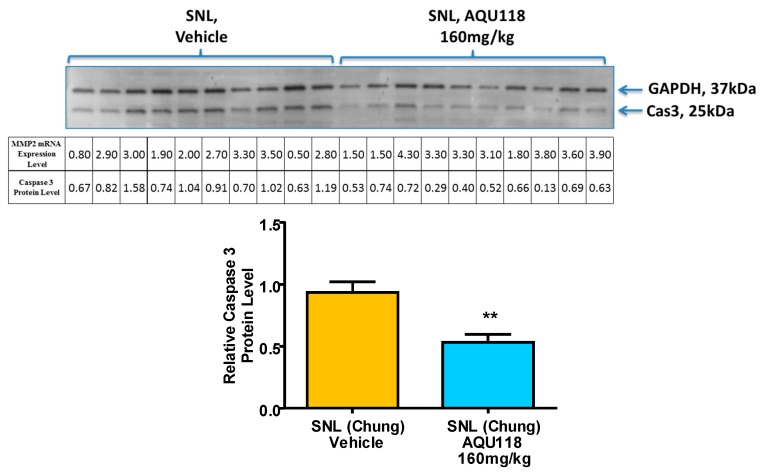
Comparison of MMP2 mRNA expression levels versus caspase-3 in a subset of ipsilateral DRG tissue for both vehicle and AQU-118 groups. Caspase 3 protein and MMP-2 mRNA expression levels were performed in the ipsilateral DRG tissue (via western blot) for both the AQU-118 (*n* = 10) and vehicle (*n* = 10) groups and compared to each other. The protein level is presented normalized to GAPDH and relative to the vehicle group (no samples were excluded in analysis). Data presented as the average ± SEM. An unpaired t-test was performed: **, *p* < 0.01.

MBP (both higher and lower molecular weight bands) and caspase-3 protein levels were measured via western blotting of tissue obtained from the left (ipsilateral) DRG of the AQU-118 group (*n* = 20) and compared with the vehicle (*n* = 20). All protein targets were normalized to glyceraldehyde-3-phosphate dehydrogenase (GAPDH) protein levels. In the samples from AQU-118 treated animals, caspase-3 levels were significantly decreased in the ipsilateral DRG but there were no significant changes observed in the levels of MBP as compared to samples obtained from vehicle-treated animals (Figure 8). The DRG samples were tested for MMP-2 mRNA expression levels and retested for caspase-3 protein levels in the AQU-118 (*n* = 10) and vehicle (*n* = 10) groups. (Figure 9). Comparison of these samples showed that the AQU-118 (*n* = 10) group exhibited a statistically significant decrease in the caspase-3 protein level as compared to the vehicle group (*n* = 10), while still exhibiting elevated MMP-2 mRNA expression levels (Figure 9).

## 3. Discussion

We have shown, for the first time, that oral dosing with AQU-118 attenuates mechanical allodynia and reduces caspase-3 protein levels in the DRG, likely via inhibition of MMP-2/-9. These findings are significant because this is the first analysis of the molecular and biochemical factors that mediate the behavioral effects seen with oral dosing with AQU-118, including the significantly reduced mechanical allodynia in both the rat SNL and CCI-IoN models of neuropathic pain as well as in the naloxone precipitated morphine withdrawal rodent model [14,15]. In this study we specifically looked at changes in biomarker levels after SNL surgery and then after dosing with AQU-118. We confirmed that after SNL surgery there is an increase in mechanical allodynia (Figure 1), which corresponds to an elevation in both the mRNA MMP-2 expression levels (Figure 3A) and caspase-3 protein levels (Figure 7A) in the DRG as compared to the sham. Once per day (SID) oral dosing with AQU-118 produces a statistically significant decrease in mechanical allodynia (Figure 1), which corresponds to a decrease in the caspase-3 protein levels (Figure 8 and Figure 9) as compared to the vehicle group.

The significant increase in MMP-2 but not MMP-9 mRNA expression levels, 25 days after SNL surgery, is consistent with the work of Ji and coworkers. They found that MMP-9 was upregulated in the DRG soon after SNL but declined several days later; this decline in MMP-9 was soon followed by a sharp increase in MMP-2 that remained elevated up to 21 days after SNL-surgery [12]. Our observation of an apparent linear correlation between the MMP-2 mRNA expression levels and that of the expression level of IL-1 β but not IL-6 (Figure 5A,B) is also consistent with the hypothesis that MMP-2 induces neuropathic pain symptoms through activation of IL-1 β and astrocyte activation [12]. However, one would also expect a concomitant elevation in the level of cleaved IL-1 β protein in the DRG which we did not observe. Instead, a significant decrease in the level of cleaved IL-1 β was observed in the ipsilateral DRG of the vehicle group as compared to the sham. (Figure 6A,B). Also, while there was an up-regulation in mRNA IL-6 in the DRG (Figure 4B), no IL-6 protein was detected [19]. Repeated attempts were made to measure IL-6 using multiple kits and reagents; while IL-6 protein was easily detected using commercially available protein standards, no IL-6 protein was measured in the DRG samples [19]. Although protein levels cannot reliably be predicted from mRNA levels, the absence of detectable IL-6 protein in the rat DRG samples in this study was surprising [20]. Several explanations could account for this result. The levels of IL-6 in the tested samples may have been below the limits of detection of the assays due to low levels of translation. Alternatively, cytokine-producing cells (i.e., glial, astrocytes & Schwann cell) are not evenly distributed throughout the DRG, which would produce variable results in quantification or detection of proteins depending upon the specific sampling location [21].

Another protein that decreased in the DRG after SNL surgery was MBP. This is consistent with the work of Shubayev and coworkers who observed a decrease in both the higher and lower molecular weight bands of MBP after L5 spinal nerve crush (SNC) [13]. They also observed that the decrease in MBP coincided with an initial increase in both MMP-9 mRNA expression and MMP-9 activity in the DRG [13]. However, while Shubayev and coworkers found that daily intraperitoneal (ip) injection of a potent broad spectrum MMP inhibitor (GM6001) was able to reverse the decrease in MBP, we saw no such reversal with oral dosing with AQU-118. Potential explanations for this disparity include the possibility that the amount of MMP inhibitor required for reversal of MBP breakdown may be higher than what was used in our current study, or that there is a significant time delay before a reversal in MBP levels occur.

While we did not observe a reversal in MBP decline after oral dosing with AQU-118, we did observe a decrease in caspase-3 protein levels in the DRG that coincided with a decrease in mechanical allodynia. This is similar to what was observed by Shubayev and coworkers, who measured a significant decrease in the level of caspase-3 in the DRG at the same time as a decrease in mechanical allodynia was noted following daily injections of GM6001 [13]. Given the fact that we observe similar decreases in both mechanical allodynia and caspase-3 protein levels, but no reversal in the levels of MBP, it is possible that either there is a significant delay between the reduction of caspase-3/apoptosis and remyelination, or that MBP breakdown is not directly connected to a caspase-3 dependent apoptotic pathway.

Previous studies have observed a strong correlation between mechanical allodynia and neuronal apoptosis [18,22,23,24,25]. For example, Sekiguki and co-workers reported that the level of mechanical allodynia observed within various surgically induced rodent models (nerve root compression versus spinal nerve compression) of neuropathic pain correlated with the level of DRG neuronal apoptosis [18]. It is well known that caspase-3 functions as the key executioner of apoptosis in neuronal cells [26,27,28]. Inhibition of caspase 3 has been found to reduce pain behaviors in rodent models of neuropathic pain [23,24,25]. Additionally, hypoxia-reoxygenation, which induces apoptosis in brain endothelial cells, also results in an increase in the expression of MMP-2, MMP-9, and caspase-3, and when these cells are exposed to a non-selective MMP inhibitor the levels of both caspase-3 and apoptosis significantly decrease [29]. This phenomenon has also been observed more recently in an in-vitro and in-vivo rat model of traumatic brain injury (TBI), supporting the idea that inhibition of MMP-2 reduces neuronal apoptosis, at least in part, through decreasing caspase-3 levels or activity [30]. These studies reinforce our findings that inhibition of MMP -2/-9 via AQU-118 reduces caspase 3 protein levels and nerve damage-induced mechanical allodynia. Clearly more studies are required to determine what intermediaries are involved in AQU-118’s ability to attenuate mechanical allodynia via caspase-3. The objective of this research is to identify biomarkers that can be used for monitoring the effects of AQU-118 in the clinic as well as to potentially screen patients with neuropathic pain who would be most likely to benefit from treatment with AQU-118.

## 4. Materials and Methods

### 4.1. Testing of Orally Administered AQU-118 in the SNL-Rat Model of Neuropathic Pain

#### 4.1.1. Animals

The use of animals was approved by the Institutional Animal Care and Use Committee (IACUC) at PsychoGenics, Inc. (IACUC 243_0214, Pain in rats, approved 9 May 2014 and IACUC 234_0314 Surgical procedures addendum for rats, approved 24 March 2014). A total of 90 male Sprague Dawley rats (200–225 g) were obtained from Envigo (Indianapolis, IN, USA). Upon receipt, rats were assigned unique identification numbers and were group housed with 3 rats per cage in polycarbonate cages with micro-isolator filter tops. All rats were examined, handled, and weighed prior to initiation of the study to assure adequate health and suitability. During the course of the study, 12/12 light/dark cycles were maintained. Rodent chow and water were provided *ad libitum* for the duration of the study.

Spinal Nerve Ligation (SNL) Chung Model [31,32]: Under general anesthesia with continuous inhalation of isoflurane, surgery was performed with aseptic procedures in a surgery unit. Sterile ophthalmic ointment was used to lubricate the eyes. Animals were observed continuously for the level of anesthesia, testing for the animal’s reflex response to a tail or paw pinch and closely monitoring the animal’s breathing. A heating pad was used to maintain body temperature at 37 °C while the animals recovered from anesthesia. The skin at the area of the lower lumber and sacral level of the rat was shaved and disinfected with betadine and alcohol. A left longitudinal incision at the level next to the vertebral column was made, and the left paraspinal muscles were separated. The transverse process of L6 was removed and nearby connective tissue cleaned to expose L5 and L6 spinal nerves [31,32]. After the nerves were isolated and clearly visualized, 4-0 silk threads were used to ligate the left L5. The muscles were sutured with 4-0 silk threads and the wound closed by staples. All rats received an analgesic (Carprofen, 5 mg/kg, subcutaneous, s.c.) immediately before and the next day after surgery. Each rat was monitored until awake and moving freely around the recovery chamber. Animals were then single-housed for the duration of the study.

#### 4.1.2. Von Frey Test for Mechanical Allodynia

Withdrawal from a mechanical stimulus was measured by applying von Frey (VF) filaments (Stoelting, Wood Dale, IL, USA) of ascending bending force to the plantar surface of the hind paws, ipsilateral and contralateral to the surgical manipulation. Filaments ranged from 0.69 to 26 g (0.692, 1.202, 1.479, 2.041, 3.63, 6, 8, 10, 15 and 26). Each filament was applied 3 times to determine withdrawal. A positive response was defined as withdrawal from the von Frey filament. Confirmation of the paw withdrawal threshold (PWT) was tested by assessing the response to the filament above and below the withdrawal response. Rats were brought to the experimental room and allowed to habituate in the room for one hour prior to testing, and acclimated to the observation chambers for 15 minutes prior to taking PWT measurements.

### 4.2. Pre-Operative Baseline Testing

Prior to surgery, all rats were tested using the VF test. Rats that had an ipsilateral PWT of less than 12 g were excluded from the study.

### 4.3. Post-Operative Testing

Two weeks following surgery, baseline VF responses were taken and animals were balanced and assigned to treatment groups based on their post-operative PWT values. Animals with a VF score over 4.5 g were excluded from the study. The animals were divided into four groups. One group (*n* = 20) which only had sham surgery was assigned as the sham group (Table 1). The next three groups consisted of those rodents that had undergone SNL surgery and were assigned as the vehicle group (*n* = 40), AQU-118 group (*n* = 20) and positive control (i.e., gabapentin) group (*n* = 10) (Table 1). Rats were dosed with either AQU-118 of vehicle (via oral gavage once per day for 5 days. Gabapentin was administered (via oral gavage) only on von Frey test days (Day 1, 3, and 5). Von Frey PWT values were measured at 60 min following compound administration (Table 1).

#### 4.3.1. Compound Formulation/Administration

AQU-118 (160 mg/kg, 0.34 mmoles/kg, once per day dosing, SID) was dissolved in 0.5% methylcellulose (400 centipoise, cps) to give a fine suspension and administered orally (P.O.). for 5 days, 60 min prior to VF testing, at a dose volume of 5 mL/kg. AQU-118 was prepared fresh daily. The vehicle (0.5% methylcellulose,400 cps) was administered at a dose volume equivalent to the test compound (5 mL/kg). Gabapentin (100 mg/kg, 0.58 mmoles/kg, SID; Toronto Research Chemicals, North York, ON, Canada) was dissolved in saline and administered P.O. on Days 1, 3, and 5 of the treatment periods, 60 min prior to VF testing, at a dose volume of 1 mL/kg. Gabapentin was prepared daily.

#### 4.3.2. Tissue Collection & Preparation

After VF test on Day 5, rats were anesthetized by CO_2_. An incision was made in the right atrium and an automated pump was used to perfuse the animal with ice cold PBS. The spinal cord (L4–L6) and DRGs (L4, L5, and L6) from both sides (into separate left and right tubes) were collected, flash frozen, and stored in a −80 °C freezer until analysis. Frozen DRG tissue samples were homogenized in RIPA buffer (75 μL per sample) using a handheld pestle homogenizer, 30 s on ice. Samples were cleared by centrifugation at 14,000 rpm for 15 min at 4 °C. Supernatants were collected and protein amount was determined using Bio-Rad DC protein kit (Biorad Laboratories, Hercules, CA, USA). For DRG samples that were analyzed by both qPCR and Western, frozen DRG samples were crushed in mini liquid nitrogen cooled mortar. Ten percent of the tissue powder was used in RNA/transcript analysis. The remaining tissue was lysed in RIPA buffer and processed as indicated above.

### 4.4. RNA and cDNA Preparation for qPCR

Sample preparation was performed via a modification of a published literature procedure [33]. DRG tissue samples were homogenized 2 × 1 min at 25 Hz in 750 μL of QIAzol Lysis Reagent with Tissue Lyser (Qiagen, Valencia, CA, USA) and 5 mm stainless steel beads (Qiagen). Disrupted samples were incubated at room temperate for 5 min. For RNA extraction, manufacturer protocol for RNeasy 96 Universal Tissue Kit (Qiagen) for RNA isolation was followed. Briefly, 150 μL of Chloroform was added and samples were shaken vigorously for 15 s followed by 3 min incubation at room temperature. The aqueous phase was separated from the organic phase by centrifugation at 6000× *g* at 4 °C for 15 min. The aqueous phase was then transferred to a new 96-well block and total RNA was precipitated with equal volume of 70% ethanol and then transferred to a RNeasy 96-well plate followed by centrifugation at 6000× *g* at room temperate for 4 min. Total RNA bound to column membranes was treated with RNase-Free DNase set (Qiagen) for 30 min, followed by 3 washing steps with RW1 and RPE buffers (provided with RNeasy 96 Universal Tissue Kit). RNA was eluted with 20 μL RNase-Free water.

RNA was quantified using Nanodrop 8000. Total RNA (0.5 μg of RNA) was reverse transcribed into cDNA with 3.2 μg random hexamers (Roche Applied Science, Indianapolis, IN, USA), 1 mM each dNTP (Roche Applied Science), 20U Protector RNase Inhibitor (Roche Applied Science), 1xTranscriptor Reverse Transcription reaction buffer and 10U Transcriptor Reverse Transcriptase (Roche Applied Science) in 20 μL total volume. The reactions were allowed to proceed at room temperature for 10 min, 55 °C for 30 min and were then inactivated at 85 °C for 5 min in a GeneAmp PCR Systems 9700 thermal cycler (Applied Biosystems, Foster City, CA, USA). cDNA samples were diluted 10-fold with RNase-Free water for qPCR analysis.

### 4.5. qPCR Analysis

All qPCR reagents and TaqMan Expression Assays were purchased from ThermoFisher Scientific (Waltham, MA, USA). Briefly, 5 μL of diluted cDNA was amplified with qPCR primer and probe sets in 1x TaqMan Fast Advanced Master Mix in 20 μL final reaction volume. Reactions were run on Applied Biosystems 7900HT Fast Block System with the following parameters: 95 °C for 20 s; 40 cycles of (95 °C/3 s; 60 °C/30 s). Each cDNA sample was run in triplicate. The Ct value for serially diluted pooled (SNL) cDNA was plotted against the log value of dilution factor and the slope of the linear regression was determined. Only assays with r2 values greater than 0.95 were used in this study. PCR efficiency was calculated as follows:

PCR Efficiency = 10 − 1/slope

The relative mRNA expression for each target was calculated following the method of Pfaffl [34]:

ΔCt_sample_ for Target = Ct Target control − Ct Target sample

ΔCt_sample_ for GAPDH = Ct GAPDHcontrol − Ct GAPDH sample

Normalizing Target to GAPDH for each sample = PCR Efficiency Target^ (ΔCtsample for Target)^/PCR Efficiency GAPDH ^(ΔCtsample for GAPDH)^

Normalized target expression was expressed relative to the average of the sham group.

### 4.6. Western Blotting

The procedure was performed following a published literature procedure [35]. IL-1β antibodies were purchased from three different vendors and validated using known standards of which only the antibody purchased from EMD Millipore passed and was used in the study. IL-6 antibodies were purchased from three different vendors of which two passed and were used in the study (R & D Systems and ThemoFisher) [19]. Caspase-3 antibody was purchased from Santa Cruz Biotechnology and MBP was purchased from Santa Cruz Biotechnology, both of which were validated and were used in the study. GAPDH was purchased from Cell Signalling Technology. Protein samples were denatured in Laemmli buffer/2-mercaptoethanol for 5 min at 95 °C. Denatured protein samples were separated by SDS-PAGE. After electrophoresis, proteins were transferred from gel to LFP-PVDF membranes by electroblotting. Non-specific binding of antibodies was blocked with 5% *w*/*v* dried milk in 1× TBST for one hour. After a brief rinse in TBST, the blots were probed with primary antibody (each diluted at 1:1000 in 1% *w*/*v* milk with 1× TBST) at 4 °C overnight. Protein-PVDF blots were washed once for 15 min followed by 3 more 5-min washes with TBST. Protein-PVDF blots were then incubated with the appropriate secondary antibody (diluted in 1:1000 prepared with 1% milk in TBST) for 1 h at room temperature. Protein-PVDF blots were washed once for 15 minutes followed by one more wash for 5 min. Antibody binding was detected using the ECL Plus Western Chemifluorescence Detection Kit (ThermoFisher Scientific). The detection solution was made fresh according to manufacturer’s directions and dispensed onto membranes. After 5 min incubation, the protein-PDVF membranes were scanned using Typhoon 9410 scanner (GE Healthcare Bioscience, Pittsburgh, PA, USA) using 457 nm blue laser for excitation and 520 nm emission filter at 400 V. The scanned images from the Typhoon were analyzed with ImageQuant TL software version 7.0 (GE Healthcare Bioscience, Piscataway, NJ, USA). Band intensities were determined using the Rolling Ball method. Each protein target was first normalized to the in-lane housekeeping protein GAPDH (from the same gel). The normalized protein target for each sample was presented as a ratio relative to the sham vehicle group.

### 4.7. Statistical Analyses

Data at all time points post-surgery were analyzed by two-way repeated measures analysis of variance (RM ANOVA) with time as the within-subjects factor and treatment as the between-subjects factor. This was followed by Fisher PLSD post-hoc comparisons where appropriate (Appendix A). Pre-surgery baseline paw withdrawal data were analyzed by one-way ANOVA. An effect was considered significant if *p* < 0.05. Data are presented as the mean ± standard error of the mean (SEM) {* *p* < 0.05, ** *p* < 0.01, *** *p* < 0.0001}.

## 5. Conclusions

The results of this study demonstrate for the first time that attenuation of mechanical allodynia via the oral dosing of the dual active MMP-2/MMP-9 inhibitor, AQU-118, in the SNL rat model of neuropathic pain is likely due to the regulation of apoptotic pathways, and specifically through reduction of caspase-3. We have confirmed previous reports that 25 days after SNL surgery, mRNA levels of MMP-2 but not of MMP-9 are elevated in the DRG. We have also been able to confirm previous reports that MBP declines and caspase-3 protein levels increase 25 days after SNL surgery. This study observed a linear relationship between levels of mRNA MMP-2 and that of IL-1β but not of IL-6. While increases in MBP or that of IL-1 β were not observed after daily oral dosing with AQU-118, their participation in attenuating mechanical allodynia cannot be ruled out at this point. While identifying a protein (caspase-3) that changes at the site of nerve damage that coincides with a reduction of mechanical allodynia is a good first step in helping elucidate a mechanism of action for AQU-118, more research will need to be performed to determine if this observation is applicable to non-rodent animal models of neuropathic pain. Additionally, while our initial focus has been on AQU-118’s effect on attenuating neuropathic pain, it would be of interest to see if other neurological disorders that implicate a caspase-3 mediated apoptotic pathway would benefit from the intervention of an orally active MMP-2/-9 inhibitor.

## Figures and Tables

**Table 1:** Protocol for SNL study using male Sprague-Dawley rats.

Group	#Rats	Route	Dose ^1,5^ (mg/kg)	Compound	Dosing Days	von-Frey Testing Days ^5^
1	20	P.O.	NA ^2^	Sham	NA ^2^	1,3,5
2	40	P.O.	NA ^3^	Vehicle ^3^	1–5	1,3,5
3	10	P.O.	100	Gabapentin	1,3,5 ^4^	1,3,5
4	20	P.O.	160	AQU-118	1–15	1,3,5

^1^ Once per day dosing via gastric gavage (P.O.), 60 min before von-Frey testing. ^2^ NA not applicable. ^3^ 0.5% methyl cellulose at a dose volume equivalent to the test compound (5 mL/kg). ^4^ Gabapentin was dissolved in saline and administered 60 min prior to von-Frey testing. ^5^ A base-line measure was taken via von-Frey before surgery and then two weeks after surgery (Day 0). The animals were then balanced and assigned to treatment groups based on their post-operative PWT values. Animals with a VF score over 4.5 g were excluded from the study.

**Figure A1:**
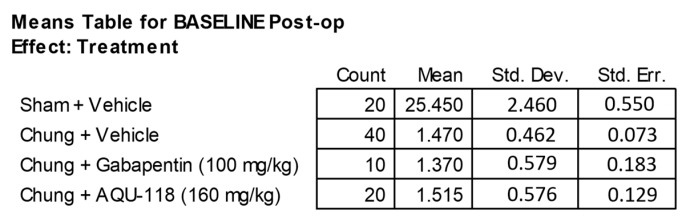
Statistical analysis of post-operative von-Frey (VF) baseline paw withdrawal threshold (PWT) of the ipsilateral hind paws. All Chung surgical groups showed a significant decrease in the PWT compared to the sham surgical group. No significant difference in post-operative baseline PWT was detected between the individual Chung surgical groups.

**Figure A2:**
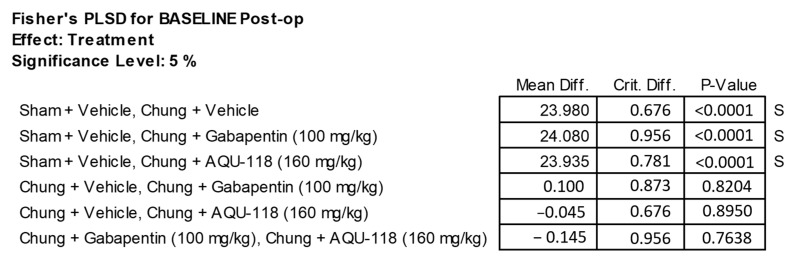
Fisher’s protected least significant difference (PLSD) test of post-operative VF baseline PWT of the ipsilateral hind paws.

**Figure A3:**
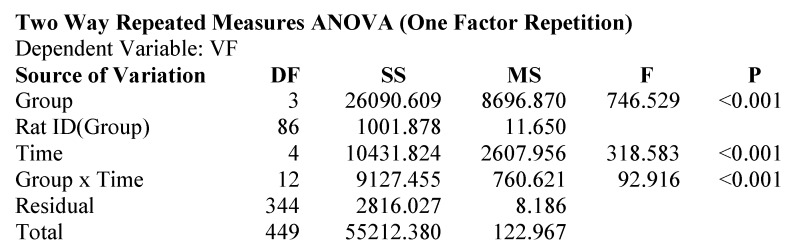
Ipsilateral Two-Way RM ANOVA statistical analysis for behavioral portion (presented in Figure 1).

**Figure A4:**
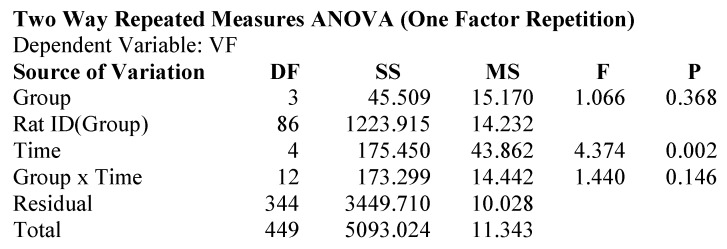
Contralateral Two-Way RM ANOVA statistical analysis for behavioral portion (presented in Figure 2).

## References

[1] Harden N., Cohen M. (2003). Unmet needs in the management of neuropathic pain. J. Pain Symptom Manag..

[2] Nightingale S. (2012). The neuropathic pain market. Nat. Rev. Drug Discov..

[3] La Fleur M., Underwood J.L., Rappolee D.A., Werb Z. (1996). Basement membrane and repair of injury to peripheral nerve: Defining a potential role for macrophages, matrix metalloproteinases, and tissue inhibitor of metalloproteinases-1. J. Exp. Med..

[4] DeLeo J.A., Colburn R.W., Nichols M., Malhotra A. (1996). Interleukin-6-mediated hyperalgesia/allodynia and increased spinal IL-6 expression in a rat mononeuropathy model. J. Interferon Cytokine Res..

[5] Sweitzer S.M., Colburn R.W., Rutkowski M., DeLeo J.A. (1999). Acute peripheral inflammation induces moderate glial activation and spinal IL-1 beta expression that correlates with pain behaviour in the rat. Brain Res..

[6] George A., Schmidt C., Weishaupt A., Toyka K.V., Sommer C. (1999). Serial determination of tumor necrosis factor-alpha content in rat sciatic nerve after chronic constriction injury. Exp. Neurol..

[7] Siebert H., Dippel N., Mader M., Frank W., Bruck W. (2001). Matrix metalloproteinase expression and inhibition after sciatic nerve axotomy. J. Neuropathol. Exp. Neurol..

[8] Schafers M., Sorkin L.S., Geis C., Shubayev V.I. (2003). Spinal nerve ligation induces transient upregulation of tumor necrosis factor 1 and 2 in injured and adjacent uninjured dorsal root ganglia in the rat. Neurosci. Lett..

[9] Mika J., Korostynski M., Kaminska D., Wawrzczak-Bargiela A., Osikowicz M., Makuch W., Przewlocki R., Przewlocka B. (2008). Interleukin-1 alpha has antiallodynic and antihyperalgesic activities in a rat neuropathic pain model. Pain.

[10] Brkic M., Balusu S., Libert C., Vandenbroucke R.E. (2015). Friends or Foes: Matrix Metalloproteinases and Their Multifaceted Roles in Neurodegenerative Diseases, Mediators of Inflammation.

[11] Tokito A., Jougasaki M. (2016). Matrix metalloproteinases in non-neoplastic disorders. Int. J. Mol. Sci..

[12] Kawasaki Y., Xu Z.Z., Wang X., Park J.Y., Zhuang Z.Y., Tan P.H., Gao Y.J., Roy K., Corfas G., Lo E.H. (2008). Distinct roles of matrix metalloproteases in the early- and late-phase development of neuropathic pain. Nat. Med..

[13] Kobayashi H., Chattopadhyay S., Kato K., Dolkas J., Kikuchi S., Myers R.R., Shubayev V.I. (2008). MMPs initiate Schwann cell-mediated MBP degradation and mechanical nociception after nerve damage. Mol. Cell Neurosci..

[14] Henry M.A., Fairchild D.D., Patil M.J., Hanania T., Hain H.S., Davis S.F., Malekiani S.A., Hu A., Sucholeiki R., Nix D., Sucholeiki I. (2015). Effect of a novel, orally active matrix metalloproteinease-2 and-9 inhibitor in spinal and trigeminal rat models of neuropathic pain. J. Oral Fac. Pain Headache.

[15] Sucholeiki I. (2012). Compounds and Methods for the Treatment of Pain and Other Disorders. U.S. Patent.

[16] Dubovy V., Brazda V., Klusakova I., Svzenska I.H. (2013). Bilateral elevation of interleukin-6 protein and mRNA in both lumbar and cervical dorsal root ganglia following unilateral chronic compression injury of the sciatic nerve. J. Neuroinflamm..

[17] Kim D.S., Figueroa K.W., Li K.W., Boroujerdi A., Yolo T., Luo Z.D. (2009). Profiling of dynamically changed gene expression in dorsal root ganglia post peripheral nerve injury and a critical role of injury-induced glial fibrillary acetic protein in maintenance of pain behaviors. Pain.

[18] Sekiguchi M., Sekiguchi Y., Konno S., Kobayashi H., Homma Y., Kikuchi S. (2009). Comparison of neuropathic pain and neuronal apoptosis following nerve root or spinal nerve compression. Eur. Spine J..

[B19-ijms-20-00811] 19.IL-6 antibodies (21 kDa) for Western blot were commercially obtained from ThermoFisher, R & D systems and Santa Cruz Biotechnology. Antibodies from R & D Systems and ThermoFisher passed validation with known controls and were then used to measure the level in DRG.

[20] Gygi S.P., Rochon Y., Franza R.B., Aebersold R. (1999). Correlation between protein and mRNA abundance in yeast. Mol. Cell. Biol..

[21] Nascimento R.S., Santiago M.F., Marques S.A., Allodi S., Martinez A.M.B. (2008). Diversity among satellite glial cells in dorsal root ganglia of the rat. Braz. J. Med. Biol. Res..

[22] Whitesides G.T., Munglani R. (2001). Cell death in the superficial dorsal horn in a model of neuropathic pain. J. Neurosci. Res..

[23] Joseph E.K., Levine J.D. (2004). Caspase signaling in neuropathic and inflammatory pain in the rat. Eur. J. Neurosci..

[24] Scholz J., Broom D.C., Youn D.-H., Mills C.D., Kohono T., Suter M.R., Moore K.A., Decosterd I., Coggeshall R.E., Woolf C.J. (2005). Blocking caspase activity prevents trans-synaptic neuronal apoptosis and the loss of inhibition in Lamina II of the dorsal Horn after peripheral nerve injury. J. Neurosci..

[25] Gradl G., Herlyn P., Finke B., Bierer P., Wree A., Witt M., Mittlmeier T., Vollmar B. (2013). A pan-caspase inhibitor reduces myocyte apoptosis and neuropathic pain in rats with chronic constriction injury of the sciatic nerve. Anesth. Analg..

[26] Elmore S. (2007). Apoptosis: A review of programmed cell death. Toxicol. Pathol..

[27] Kavanagh E., Rodhe J., Burguillos M.A., Venero J.L., Joseph B. (2014). Regulation of caspase-3 processing by cIAP2 controls the switch between pro-inflammatory activation and cell death in microglia. Cell Death Dis..

[28] Amelio M.D., Cavallucci V., Cecconi F. (2010). Neuronal caspase-3 signaling: Not only cell death. Cell Death Differ..

[29] Lee S.R., Lo E.H. (2004). Induction of caspase-mediated cell death by matrix metalloproteinases in cerebral endothelial cells after hypoxia-reoxygenation. J. Cereb. Blood Flow Metab..

[30] Abdul-Muneer P.M., Conte A.A., Haldar D., Long M., Patel R.K., Santhakumar V., Overall C.M., Pfister B.J. (2017). Traumatic brain injury induced matrix metalloproteinase 2 cleaves CXCL12α(stromal cell derived factor 1α) and causes neurodegeneration. Brain Behav. Immun..

[31] Kim S.H., Chung J.M. (1992). An experimental model for peripheral neuropathy produced by segmental spinal nerve ligation in the rat. Pain.

[32] Chung J.M., Kim H.K., Chung K. (2004). Segmental spinal nerve ligation model of neuropathic pain. Methods Mol. Med..

[33] Menalled L.B., Kudwa A.E., Miller S., Fitzpatrick J., Watson-Johnson J., Keating N., Ruiz M., Mushlin R., Alosio W., McConnell K. (2012). Comprehensive behavioral and molecular characterization of a new knock-in mouse model of Huntington’s disease: zQ175. PLoS ONE.

[34] Pfaffl M.W. (2001). A new mathematical model for relative quantification in real-time RT–PCR. Nucleic Acids Res..

[35] Menalled L.B., Kudwa A.E., Oakeshott S., Farrar A., Paterson N., Filippov I., Miller S., Kwan M., Olsen M., Beltran J. (2014). Genetic deletion of transglutaminase 2 does not rescue the phenotypic deficits observed in R6/2 and zQ175 mouse models of Huntington’s disease. PLoS ONE.

